# Supplementary material for: Dietary Intake, Feeding Pattern, and Nutritional Status of Children with Cerebral Palsy in Rural Bangladesh
Source: Nutrients. 2023 Sep 29;15(19):4209. doi: 10.3390/nu15194209 (PMC10574640; doi:10.3390/nu15194209)
Supplement: Supplementary file 1 [file nutrients-15-04209-s001.zip › nutrients-2605057-supplementary.pdf]

nutrients MDPI

Table S1: Socio-demographic and clinical characteristics of participating children

Characteristics	n (%)	Normal weight-for-age, n (%)	Underweight, n (%)	p value[3]
**Age in years, n=74[1]**				
Mean (SD)	3.6 (2.7)	2.6 (1.8)	4.1 (2.8)	0.02
<2	15 (20.3)	7 (26.9)	4 (13.3)	0.17[4]
2-5	39 (52.7)	16 (61.5)	17 (56.7)	
6-11	20 (27.0)	3 (11.5)	9 (30.0)	
**Sex, n=75**				
Female	32 (42.7)	10 (38.5)	15 (50.0)	0.39
Male	43 (57.3)	16 (61.5)	15 (50.0)	
**Type of accommodation, n=69[1]**				
Temporary shelter (jhupri)	0 (0.0)	0 (0.0)	0 (0.0)	0.37[4]
Mud houses (kutcha house)	35 (50.7)	12 (48.0)	17 (58.6)	
Semi-permanent (semi-pucca) house	27 (39.1)	12 (48.0)	9 (31.0)	
Permanent brick (pucca) house	7 (10.1)	1 (4.0)	3 (10.3)	
**Source of drinking water, n=69[1]**				
Tube-well	66 (95.7)	24 (96.0)	28 (96.6)	0.91[4]
Tap water	3 (4.3)	1 (4.0)	1 (3.4)	
**Type of toilet use, n=69[1]**				
Non-sanitary	24 (34.8)	7 (28.0)	14 (48.3)	0.13
Sanitary	45 (65.2)	18 (72.0)	15 (51.7)	
**Mother's education level, n=69[1]**				
No formal schooling	2 (2.9)	1 (4.0)	1 (3.4)	0.72[4]
Received formal schooling	67 (97.1)	24 (96.0)	28 (96.6)	
**Mother's occupation, n=69[1]**				
Not involved in any IGA	64 (92.8)	23 (92.0)	28 (96.6)	0.44[4]
Involved in IGA	5 (7.2)	2 (8.0)	1 (3.4)	
**Monthly family income (BDT, USD), n=68[1]**				
≤10000	36 (52.9)	11 (45.8)	16 (55.2)	0.44[4]
10001-20000	28 (41.2)	12 (50.0)	10 (34.5)	
20001-30000	4 (5.9)	1 (4.2)	3 (10.3)	
**Presence of associated impairment[2]**				
Epilepsy	23 (30.7)	11 (45.8)	10 (34.5)	0.40
Intellectual impairment	26 (34.7)	15 (71.4)	15 (57.7)	0.33
Visual impairment	8 (10.7)	5 (20.0)	3 (10.3)	0.27[4]
Hearing impairment	7 (9.3)	4 (16.7)	3 (10.3)	0.39[4]
Speech impairment	47 (62.7)	15 (60.0)	21 (72.4)	0.25
**Presence of swallowing difficulties, n=69[1]**				
No	60 (86.9)	22 (88.0)	25 (86.2)	0.58[4]
Yes	9 (13.0)	3 (12.0)	4 (13.8)	
**Presence of reflux, n=69[1]**				
No	61 (88.4)	23 (92.0)	25 (86.2)	0.41[4]
Yes	8 (11.6)	2 (8.0)	4 (13.8)	
**Predominant motor type and topography, n=75[1]**				
Spastic	62 (82.7)	23 (88.5)	24 (80.0)	0.78[4]
Dyskinesia	4 (5.3)	1 (3.8)	2 (6.7)	
Ataxia	3 (4.0)	1 (3.8)	1 (3.3)	
Hypotonia	6 (8.0)	1 (3.8)	3 (10.0)	
**Topography**				
Unilateral	8 (12.9)	4 (17.4)	2 (8.3)	0.31[4]
Bilateral	54 (87.1)	19 (82.6)	22 (91.7)	
**GMFCS level, n=72[1]**				
I-II	11 (15.3)	2 (8.0)	4 (13.3)	0.43[4]
III-V	61 (84.7)	23 (92.0)	26 (86.7)	

Characteristics	n (%)	Normal weight-for-age, n (%)	Underweight, n (%)	p value[3]
**MACS level, n=74[1]**				
I-II	19 (25.7)	9 (34.6)	7 (23.3)	0.26
III-V	55 (74.3)	17 (65.4)	23 (76.7)	

[1]Missing data; [2]n=68 for epilepsy, n=61 for intellectual impairment, n=69 for visual impairment, n=68 for hearing impairment, n=69 for speech impairment. [3]chi-squared test; [4]Fisher's exact test
